# Dietary intake from complementary feeding is associated with intestinal barrier function and environmental enteropathy in Brazilian children from the MAL-ED cohort study

**DOI:** 10.1017/S0007114520000215

**Published:** 2020-01-22

**Authors:** P. N. Costa, A. M. Soares, J. Q. Filho, F. S. Junior, R. Ambikapathi, E. T. Rogawski McQuade, R. L. Guerrant, L. E. Caulfield, A. A. M. Lima, B. L. L. Maciel

**Affiliations:** 1 Nutrition Post-Graduation Program, Department of Nutrition, Federal University of Rio Grande do Norte, Natal, Rio Grande do Norte, Brazil; 2 Department of Physiology and Pharmacology, INCT – Instituto de Biomedicina do Semiárido Brasileiro (IBISAB), Federal University of Ceará, Fortaleza, Ceará, Brazil; 3 Department of Public Health, Purdue University, West Lafayette, IN, USA; 4 Department of Public Health Sciences, University of Virginia, Charlottesville, VA, USA; 5 Center for Global Health, Division of Infectious Diseases and International Health, University of Virginia School of Medicine, Charlottesville, VA, USA; 6 Center for Human Nutrition, Department of International Health, Johns Hopkins Bloomberg School of Public Health, Baltimore, MD, USA

**Keywords:** Infants, Nutrient intake, Intestinal permeability, Biomarkers, Intestinal infections

## Abstract

A child’s diet contains nutrients and other substances that influence intestinal health. The present study aimed to evaluate the relations between complementary feeding, intestinal barrier function and environmental enteropathy (EE) in infants. Data from 233 children were obtained from the Brazilian site of the Etiology, Risk Factors, and Interactions of Enteric Infections and Malnutrition and the Consequences for Child Health and Development Project cohort study. Habitual dietary intake from complementary feeding was estimated using seven 24-h dietary recalls, from 9 to 15 months of age. Intestinal barrier function was assessed using the lactulose–mannitol test (L–M), and EE was determined as a composite measure using faecal biomarkers concentrations – α-1-antitrypsin, myeloperoxidase (MPO) and neopterin (NEO) at 15 months of age. The nutrient adequacies explored the associations between dietary intake and the intestinal biomarkers. Children showed adequate nutrient intakes (with the exception of fibre), impaired intestinal barrier function and intestinal inflammation. There was a negative correlation between energy adequacy and L–M (*ρ* = −0·19, *P* < 0·05) and between folate adequacy and NEO concentrations (*ρ* = −0·21, *P* < 0·01). In addition, there was a positive correlation between thiamine adequacy and MPO concentration (*ρ* = 0·22, *P* < 0·01) and between Ca adequacy and NEO concentration (*ρ* = 0·23; *P* < 0·01). Multiple linear regression models showed that energy intakes were inversely associated with intestinal barrier function (*β* = −0·19, *P* = 0·02), and fibre intake was inversely associated with the EE scores (*β* = −0·20, *P* = 0·04). Findings suggest that dietary intake from complementary feeding is associated with decreased intestinal barrier function and EE in children.

Infancy is a crucial period for the development of the gastrointestinal tract and gut microbiota, as well as the immune system^(1,2)^. Early nutrition is one of the prime factors to regulate the complex relationship of gastrointestinal tract–microbiota–immune maturation^(2–4)^. Many nutrients and dietary components can directly modulate the intestinal barrier function^(5–8)^. This modulation is mainly done by intestinal microbiota and immune system cross-talk, as previously demonstrated in experimental and human studies^(9–11)^. The Brazilian Ministry of Health^(12)^, by following the WHO guidelines^(13,14)^, recommends exclusive breast-feeding from birth to 6 months of life and, after this period, a nutritionally adequate and safe complementary feeding associated with continued breast-feeding up to 24 months, even though breast-feeding may continue beyond 2 years.

Although breast milk composition and its influence on the immune system and protection to infectious diseases have been extensively studied, few studies have attempted to analyse the impact of nutrient intake from complementary feeding on these same variables. Complementary feeding has a potential influence on intestinal development and modulation, considering the roles of the diet in intestinal barrier function protection^(6,7,15,16)^.

Dietary components including macronutrients, vitamins and minerals are regulators of the intestinal barrier function. These components include, for example, the amino acid glutamine that maintains intestinal integrity, suppresses pro-inflammatory signalling pathways and protects cells against apoptosis and cellular stresses^(8,17)^. In children, glutamine has been shown to improve intestinal barrier function and nutritional status, with better results in the urinary biomarkers of intestinal barrier function^(18–20)^. Fibres are another essential dietary component that may modulate gut barrier function and immunity^(6)^. Most of these benefits have been associated with the SCFA, the end products of fermentation of dietary fibres by the intestinal microbiota^(21)^. SCFA are essential for protection and repair^(21)^ and have anti-inflammatory^(15)^ effects on the intestinal barrier function. However, dietary fibres may also affect enteric immunity directly, acting in the inflammatory pathway in immune^(22)^ and intestinal epithelial cells^(7)^.

The onset of complementary feeding coincides with an increased incidence of enteric infections among children^(23)^. Repeated enteric infections, even when subclinical, can lead to intestinal barrier dysfunction, resulting in a chronic condition known as environmental enteropathy (EE)^(24)^. EE is thought to primarily affect children living in low- and middle-income countries, with limited access to quality food, improved water, sanitation and hygiene^(25,26)^. EE is characterised as the adverse outcome of frequent and numerous subclinical or clinical enteric infections, resulting in a state of persistent immune activation and altered intestinal barrier function^(24)^. Among its outcomes, EE has been hypothesised to underlie high rates of stunting and growth failure^(27,28)^, inadequate response to vaccines^(29)^ and impaired cognitive development^(30)^. Recent data indicate that EE biomarkers are influenced by enteropathogen burden, sanitation and hygiene conditions and socio-economic status (SES)^(23,26,31)^. However, it is not clear if and to what extent EE could be additionally influenced by factors other than enteropathogens, such as dietary intake.

Despite the potential role of dietary components on the modulation of intestinal barrier function and the outcomes of EE, few studies have sought to understand the role of complementary foods in intestinal function and EE using a prospective cohort approach. The present study aimed to evaluate the relations between complementary feeding, intestinal barrier function and EE in children. The present study hypothesis is that the dietary intake from complementary feeding is associated with the biomarkers of intestinal barrier function and EE.

## Methods

### Study population

The present study is part of the Etiology, Risk Factors, and Interactions of Enteric Infections and Malnutrition and the Consequences for Child Health and Development Project (MAL-ED) 5-year cohort study, with enrolment beginning November 2009 and final follow-up ending February 2017^(32)^. The Ethics Committee of the Federal University of Ceará and the University of Virginia approved the study protocols, and mothers or guardians only enrolled the infants in the study after authorisation.

The study was conducted in the community of Parque Universitário, located in Fortaleza, capital of Ceará state, northeastern Brazil, with approximately 33 000 inhabitants of families with low income, as previously described^(33)^. The present study enrolled 233 children. Inclusion criteria were healthy newborns up to 17 d old, parents/caregiver for the child who had no plans to leave the study area for at least 6 months from enrolment in the study and caregiver’s willingness and availability to be visited at home twice a week. The exclusion criteria were if the family had plans to be moving from the recruitment area in a period longer than 30 consecutive days during the first 6 months of follow-up; maternal age <16 years; mother with another child enrolled in the study; twin pregnancy; severe illness requiring hospitalisation or any other severe or chronic condition such as HIV, tuberculosis, kidney disease, chronic heart failure or severe liver disease and birth weight <1500 g^(32,33)^.

### Data collection

Infant breast-feeding practices and the introduction of non-breast milk liquids (water, juice, teas and other milks) information were collected during twice-weekly surveillance visits^(34)^. From 0 to 8 months, the breast-feeding practices were obtained by twice-weekly and monthly questionnaires. Also, from 9 to 15 months, food intake was assessed by monthly 24-h food recall. Breast-feeding was categorised as exclusive (if the child received only breast milk, except for drops or syrups containing vitamins, mineral supplements or medicines), predominant (if the child consumed water or other water-based liquids in addition to breast milk), partial (if the child received other types of milk, formula and/or solid/semi-solid foods, in addition to breast milk) or none, according to the definitions of WHO^(35)^ and Patil *et al.*^(36)^. From the weekly collections, we also analysed the sum of days of exclusive breast-feeding.

A questionnaire including variables about water source and sanitation facilities, household assets, maternal education and monthly household income investigated the SES. The variables in the questionnaire were combined to form a water, assets, maternal education and household income (WAMI) index^(37)^. The WAMI index ranges in values from 0 to 1, where 0 represents the lowest possible SES and 1 the highest.

### Dietary intake assessment

The habitual dietary intake was determined by collecting quantitative 24-h recalls, applied monthly from 9 to 15 months of the child, according to the methodology described by Caulfield *et al.*^(34)^. Trained clinical research staff collected the dietary intake information. The research staff completed the forms by hand. Other local research staff reviewed the forms and entered the information into a computer. Researchers from the Johns Hopkins University Bloomberg School of Public Health conducted multiple searches to identify errors and analysed recipes to obtain nutrient information. Re-trainings of field staff occurred based on error identification. Communication with the researchers at Johns Hopkins University Bloomberg School of Public Health resolved issues or questions identified during data collection. The dietary intake data were converted to nutrients using locally adapted food composition tables^(38,39)^ and the United States Department of Agriculture^(40)^ database. The analysis also included manufacturers’ information on industrial products, when necessary.

### Nutritional adequacy from complementary feeding

The Kolmogorov–Smirnov test verified the normality of nutrients data. Nutrients with asymmetric distribution were square rooted and had the symmetry tested again. After conversion to square root, PUFA, vitamin B_2_, vitamin B_6_, vitamin B_12_ and vitamin C remained with asymmetric distribution and were not included in the analysis. Because of the day-to-day variation in food consumption, the method developed by Nusser *et al.*^(41)^ to adjust for intra-individual variability was used. One-way ANOVA estimated the within- and between-person variance based on the quadratic means from the ANOVA. Then, back-transformation of the corrected square rooted values to return the estimated usual intakes to their original scale was done. To control for confounding factors inherent in total energy intake and to reduce the errors associated with dietary measurements, nutrient intake was adjusted for energy using the residual method and data were transformed back to their original units^(42)^.

From the seven 24-h food recalls per child, the nutritional adequacies for energy, macronutrients and fibre were calculated as the ratio of a child’s median daily intake:the recommended intake. The energy adequacy ratios were calculated considering the median energy intake from 9 to 15 months and the recommendation of 335 kJ/kg per d, considering the child’s weight at 15 months^(43)^. For carbohydrate and protein adequacies ratios, the median nutrient intake was divided by the RDA^(44)^. For fibre adequacy ratio, the median fibre intake was divided by the adequate intakes^(44)^.

Nutrient adequacy ratios (NAR) were calculated as the median micronutrient intake divided by the RDA^(45,46)^ to describe the micronutrient adequacy of complementary feeding. Age- and sex-specific RDA were used based on the recommendation developed by the Institute of Medicine^(47–49)^. The mean adequacy ratio (MAR) was used as an index of the overall nutritional quality of the complementary feeding^(50)^. The MAR was calculated from the sum of NAR of vitamin A, thiamine, folate, Ca, Fe and Zn divided by the total number of nutrients (six micronutrients, in our case). In the present study, the NAR and MAR were not truncated to 1, considering that most of the nutrients presented intakes higher than the recommendations and that these would give important variability to correlate with the intestinal biomarkers assessed. Therefore, a MAR of 1·00 or higher indicated an intake equal or greater to the recommended value for all nutrients, and on the other hand, a MAR below 1·00 indicated less than the recommended intake for one or more nutrients.

### Intestinal barrier function and environmental enteropathy

The intestinal barrier function and inflammation were evaluated from urine and non-diarrheal faecal samples of the children, following the methodology described by Kosek *et al.*^(27)^. The lactulose:mannitol (L–M) test at 15 months old evaluated the intestinal barrier function (permeability and absorption capacity) of the children. In brief, the children should be fasted for 2 h and empty bladder prior to administration of a solution containing lactulose (250 mg/ml) and mannitol (50 mg/ml) at a dose of 2 ml/kg (maximum administered dose, 20 ml) at a concentration of 1002 mOsm/l. The child’s total urine for 5 h after the solution administration was recorded, and one to two drops of chlorhexidine (2·35 %) were added to the urine samples. Samples were aliquoted and stored at −80°C until testing. The lactulose and mannitol concentrations were measured by HPLC with pulsed amperometric detection, following the methodology proposed by Barbosa *et al.*^(51)^. Three proteins were measured from the stool at 15 months: *α*-1-antitrypsin (AAT), as a biomarker of protein loss; neopterin (NEO), as a biomarker of T helper type 1 (Th1) immune cell activation; and myeloperoxidase (MPO), which is indicative of neutrophilic activity in the intestinal mucosa. Stool samples were collected in a sterile container, aliquoted and stored in cryotubes at −20°C until analysis. Samples were evaluated using ELISA kits for AAT (Biovendor), MPO (Alpaco) and NEO (GenWay Biotech), according to the manufacturer’s instructions.

A simplified score based on the percentile category of AAT, MPO and NEO was used to indicate EE, as described by Kosek *et al.*^(27)^. Briefly, these faecal biomarkers were combined into a single score, which was able to indicate EE and predicted less length gained between children^(27)^. Faecal markers’ concentrations were categorised based on the distribution of all measurements: as 0 (≤25th percentile), 1 (25–75th percentile) or 2 (≥75th percentile). Therefore, the EE score ranged in values from 0 to 10. The formula used for the composite EE score was as follows: EE score = 2 × (AAT category) + 2 × (MPO category) + 1 × (NEO category).

### Pathogen burden

Non-diarrhoeal stool samples were collected monthly and tested for twenty-nine enteropathogens using quantitative PCR with custom-designed TaqMan Array Cards (ThermoFisher), as previously described^(52,53)^. A sample was positive for a pathogen when the quantitative PCR cycle threshold was less than thirty-five (the analytic limit of detection). The total number of bacteria, viruses and parasites detected in each stool sample was calculated in quantification cycles (Cq), and the average number of each pathogen group between 9 and 15 months of age was used to characterise subclinical pathogen burden during this age period. These pathogens were grouped as follows: bacteria (*Campylobacter* spp., *Shigella*, enteroaggregative *Escherichia coli*, typical enteropathogenic *E. coli*, atypical enteropathogenic *E. coli*, enterotoxigenic *E. coli*, Shiga toxin-producing *E. coli*, *Aeromonas*, *Helicobacter pylori*, *Plesiomonas*, *Salmonella* and *Vibrio cholerae*); viruses (rotavirus, norovirus, adenovirus 40/41, astrovirus and sapovirus) and parasites (*Cryptosporidium*, *Giardia*, *Enterocytozoon bieneusi*, *Trichuris*, *Enterocytozoon intestinalis*, *Cyclospora*, *Isospora*, *Entamoeba histolytica*, *Acyclostoma*, *Ascaris*, *Necator* and *Strongyloides*).

### Statistical analysis

Data were double entered by trained personnel, with consistency checks and data cleaning. The Kolmogorov–Smirnov test checked the normality of data distribution. Continuous variables with symmetrical distributions were presented as means and standard deviations; variables with skewed distribution, as median and range (Q1–Q3); and categorical variables, as absolute and relative frequencies.

Spearman’s rank correlation coefficients (*r*^2^) assessed the relationship between the nutrient adequacies and intestinal barrier function (L–M test results) and EE biomarkers. Two multiple linear regression methods were constructed considering (1) intestinal barrier function using the L–M test and (2) the EE score as dependent variables. These variables were tested for normality, and the L–M test results were normalised using *z*-scores. SES, measured using the WAMI index, was included in the final models as an adjustment variable. Exclusive breast-feeding duration could have long-term positive effects on intestinal parameters and was included in the models as an adjustment variable. Because the L–M and EE are affected by enteropathogens, bacterial, viral and parasites burdens, these variables were included in the final models. Nutrient intake variables were the independent variables, and these were inserted into the adjusted models. Independent variables in the final models were selected considering multicollinearity, and those showing *r* > 0·7 were excluded. Also, tolerance values over 0·10 and variance inflation factors less than 10 were considered to select independent variables in the models. Residuals scatterplots were also used to check outliers, normality, linearity, homoscedasticity and independence of residuals to select final models. Thus, final models’ independent variables were nutrient adequacy for energy, protein and fibre, and MAR. The results of the regression models were presented with *β*-coefficients, 95 % CI and *P* values. The statistical software SPSS version 23.0 for Windows was used.

Lima *et al.*^(20)^, in a previous prospective double-blinded randomised placebo-controlled trial with children from the same locality of our study, calculated sample size using the lactulose:mannitol ratio as the primary outcome. The sample size was of forty-two children with 90 % power to detect a reduction in 30 % or more in lactulose:mannitol ratios at a significance level of *P* = 0·05. In this previous study, the mean lactulose:mannitol ratio used was 0·13 ± 0·04, similar to those of our children. In the present study, the sample size was determined by the overall feasibility of data from the MAL-ED study, which was not explicitly addressed to quantifying dietary aspects. A commonly used sample size criterion for developing a prediction model is the events per variables criterion, in particular events per variable ≥ 10. However, recent publications have shown that events per variable is not an appropriate criterion for binary prediction model studies^(54)^. For these reasons, the available number of children and events per variable were used without making any statements about power.

## Results

During the follow-up of the present study, 61·5 % of the children were still breastfed to some degree at 15 months of age. The median of accumulated days of exclusive breast-feeding was about 2·5 months (76 d), during the first 6 months of life. The median number of assets was 7 (out of 8 assessed). The median WAMI score was 0·8 (0·8, 0·9), and all participants had access to improved water and sanitation. The median lactulose:mannitol was of 0·09 (0·05–0·14), and the median EE score was of 5·0 (3·0–7·0). Considering the pathogen burden, bacteria presented the highest Cq from 9 to 15 months of age, of 1·00 (0·75–1·40) (Table 1).

**Table 1.** General characteristics of children (*n* 233) in the Brazilian MAL-ED cohort site (Medians and 25th and 75th percentiles (Q1, Q3); numbers and percentages)

Categorical variables	*n*	%
Male children	118	50·6
Breastfed children at 15 months	143	61·5
Continuous variables	Median	Q1, Q3
Exclusive breast-feeding (d)*	76	38, 126
Socio-economic status		
Number of assets	7	7, 7
Maternal education (years)	9	8, 12
Monthly household income (USD)†	334·3	304·3, 393·4
WAMI	0·8	0·8, 0·9
Pathogen burden‡		
Bacteria (Cq)	1·00	0·75, 1·40
Viruses (Cq)	0·20	0·00, 0·33
Parasites (Cq)	0·00	0·00, 0·20
Intestinal barrier function		
% Lactulose excretion	0·14	0·08, 0·24
% Mannitol excretion	1·86	0·77, 3·02
Lactulose:mannitol	0·09	0·05, 0·14
Intestinal inflammation biomarkers		
*α*-1-Antitrypsin (ng/ml)	12·2	6·5, 18·6
Myeloperoxidase (ng/ml)	5074	2130, 1190
Neopterin (nmol/l)	1368	778, 3163
EE score	5·0	3·0, 7·0

MAL-ED, Etiology, Risk Factors, and Interactions of Enteric Infections and Malnutrition and the Consequences for Child Health and Development Project; USD, US dollars; WAMI, Water, Assets, Mother’s education and Income index; Cq, mean quantification cycle; EE, environmental enteropathy.

* Presented as the sum of accumulated days of exclusive breast-feeding during the first 6 months of life.

† Presented as USD using the exchange rate from 1 January 2010.

‡ Calculated as Cq from 9 to 15 from months of bacteria (*Campylobacter* spp., *Shigella*, enteroaggregative *Escherichia coli*, typical enteropathogenic *E. coli*, atypical enteropathogenic *E. coli*, enterotoxigenic *E. coli*, Shiga toxin-producing *E. coli*, *Aeromonas*, *Helicobacter pylori*, *Plesiomonas*, *Salmonella* and *Vibrio cholerae*); viruses (rotavirus, norovirus, adenovirus 40/41, astrovirus and sapovirus) and parasites (*Cryptosporidium*, *Giardia*, *Enterocytozoon bieneusi*, *Trichuris*, *Enterocytozoon intestinalis*, *Cyclospora*, *Isospora*, *Entamoeba histolytica*, *Acyclostoma*, *Ascaris*, *Necator* and *Strongyloides*).

A total of 1234 dietary 24-h recalls were collected. Energy and all macronutrients intakes were in accordance with the recommended values (Table 2). However, fibre intake (3·1 g/d) was below adequate intake (19 g/d), with a ratio of 0·2 (Table 2). Micronutrients habitual intakes were high to all vitamins (vitamin A, thiamine and folate) and minerals (Fe, Zn and Ca) analysed. These results lead to an MAR of 2·20 in the complementary feeding of the study children (Table 2).

**Table 2.** Nutrient intakes and mean nutrient adequacy from complementary food intake, in children from 9 to 15 months (*n* 233) in the Brazilian MAL-ED cohort site (Recommended dietary allowances; medians and 25th and 75th percentiles (Q1, Q3); adequacy)

	RDA^(55)^	Intake*		Adequacy
		Median	Q1, Q3	
Energy (kJ/kg per d)	335†	333·9	253·9, 416·7	1·0‡
Fibre (g/d)	19§	3·1	2·3, 4·5	0·2‡
Macronutrients				
Protein (g/d)	13	36·9	34·3, 40·2	2·8‡
Carbohydrate (g/d)	130	120·3	114·6, 124·8	0·9‡
Total fat (g/d)	–	28·6	26·8, 30·3	–
Saturated fat (g/d)‖	–	16·2	4	–
Monounsaturated fat (g/d)‖	–	7·5	1·2	–
Cholesterol (mg/d)	–	116·2	104·1, 133·1	–
Sugar (g/d)	–	26·8	19·3, 33·9	–
Micronutrients¶				
Vitamin A (μg/d)	300	950·1	855·4, 1065·2	3·3
Thiamin (mg/d)	0·5	0·7	0·6, 0·8	1·4
Folate (μg/d)	150	154	134, 177	1·1
Fe (mg/d)	7	14	11, 15·7	1·9
Zn (mg/d)	3	8·3	6·9, 9·8	2·7
Ca (mg/d)	340	965·2	900·2, 1054·4	2·8
MAR**	–	–	–	2·2

MAL-ED, Etiology, Risk Factors, and Interactions of Enteric Infections and Malnutrition and the Consequences for Child Health and Development Project; MAR, mean adequacy ratio; NAR, nutrient adequacy ratio calculated as the mean daily intake/RDA.

* Nutrients intakes were adjusted according to the individual energy intake^(42)^ and intra-individual variability^(41)^.

† Energy recommendation, according to FAO/WHO/United Nations University (2004)^(43)^.

‡ Adequacy ratio calculated as the daily intake/RDA.

§ Presented as adequate intakes.

‖ Presented as mean value and standard deviation.

¶ Presented as nutrient adequacy ratio, calculated as the mean daily intake/RDA.

** MAR calculated as ΣNAR/6.

When testing the correlations between energy and nutrient adequacies with the intestinal barrier function and faecal biomarkers (Table 3), a negative correlation between the energy adequacy ratio and the L:M ratio *z-*score was found (*r*^2^ −0·19; *P* < 0·05). A negative correlation between folate NAR and NEO concentrations was also observed (*r*^2^ −0·21; *P* < 0·01), and thiamine and Ca NAR presented a positive correlation with MPO (*r*^2^ 0·22, *P* < 0·01) and NEO (*r*^2^ 0·23, *P* < 0·01), respectively (Table 3).

**Table 3.** Spearman’s rank correlation coefficient (*r*^2^) between nutrient adequacy and intestinal barrier function and faecal biomarkers

Nutrient adequacy	%Lac	%Man	LM	MPO	NEO	AAT	EE score
	*r*^2^						
Energy ratio	0·00	0·13	−0·19*	−0·10	−0·10	−0·11	−0·14
Carbohydrate ratio	0·00	0·00	−0·04	−0·03	−0·16	0·02	−0·06
Fibre ratio	0·11	0·13	−0·07	−0·12	−0·12	−0·06	−0·07
Protein ratio	0·11	0·10	0·00	−0·03	0·02	0·04	0·03
NAR vitamin A	0·02	0·00	0·02	0·02	−0·01	−0·03	−0·02
NAR thiamin	0·03	0·02	−0·01	0·22**	0·08	−0·06	0·11
NAR folate	−0·03	−0·12	0·12	0·03	−0·21**	0·03	−0·02
NAR Fe	−0·06	−0·06	0·04	0·12	0·05	−0·01	0·06
NAR Zn	0·03	0·03	−0·01	0·11	0·03	−0·10	0·00
NAR Ca	−0·03	−0·08	0·11	0·06	0·23**	−0·06	0·01
MAR	−0·03	−0·05	0·03	0·11	0·06	−0·06	0·02

%Lac, percentage of lactulose urinary excretion; %Man, percentage of urinary excretion of mannitol; LM, ratio of the urinary excretion of lactulose:mannitol; MPO, myeloperoxidase; NEO, neopterina; AAT, *α*-1-antitrypsin; EE score, environmental enteropathy score; NAR, nutrient adequacy ratio calculated as the mean daily intake/RDA; MAR, mean adequacy ratio calculated as ΣNAR/6.

* *P* < 0·05, ** *P* < 0·01.

The intestinal barrier function multiple linear regression model showed that the higher the energy intake the lower the L:M *z*-scores (*β* = −0·20, *P* = 0·01) (Table 4). For EE, the multiple linear regression model showed an inverse association with the fibre adequacy ratio (*β* = −0·19, *P* = 0·04).

**Table 4.** Multiple linear regression for associations of habitual dietary intake from complementary feeding and intestinal barrier function and environmental enteropathy (*β*-Coefficients and 95 % confidence intervals)

	Lactulose:mannitol *z*-score			EE score		
Constant B...	1·64			8·22		
Variables	*β*	95 % CI	*P*	*β*	95 % CI	*P*
Bacterial burden	0·20	0·10, 0·74	0·01	−0·02	−1·00, 0·80	0·83
Virus burden	−0·02	−0·88, 0·65	0·78	0·24	0·68, 4·96	0·01
Parasites burden	0·06	−0·59, 0·29	0·50	−0·02	−1·32, 1·13	0·87
WAMI index	−0·28	−4·79, −1·18	0·001	−0·16	−9·40, 0·71	0·09
Days exclusively breastfed*	−0·28	−0·00, 0·00	0·84	0·09	−0·04, 0·01	0·34
Energy ratio	−0·20	−0·98, −0·11	0·01	−0·13	−2·04, 0·38	0·18
Protein ratio	0·14	−0·06, 0·66	0·10	0·12	−0·38, 1·64	0·22
Fibre ratio	−0·07	−1·84, 0·74	0·40	−0·19	−7·23, −0·03	0·04
Mean adequacy ratio	0·05	−0·29, 0·57	0·53	−0·06	−1·56, 0·85	0·56

EE, environmental enteropathy; WAMI index, water, assets, maternal education and household income index.

* Presented as the sum of the days of exclusive breast-feeding during the first 6 months of life. Pathogen burdens calculated as mean quantification cycle (Cq) from 9 to 15 months of bacteria (*Campylobacter* spp., *Shigella*, enteroaggregative *Escherichia coli*, typical enteropathogenic *E. coli*, atypical enteropathogenic *E. coli*, enterotoxigenic *E. coli*, Shiga toxin-producing *E. coli*, *Aeromonas*, *Helicobacter pylori*, *Plesiomonas*, *Salmonella* and *Vibrio cholerae*); viruses (rotavirus, norovirus, adenovirus 40/41, astrovirus and sapovirus) and parasites (*Cryptosporidium*, *Giardia*, *Enterocytozoon bieneusi*, *Trichuris*, *Enterocytozoon intestinalis*, *Cyclospora*, *Isospora*, *Entamoeba histolytica*, *Acyclostoma*, *Ascaris*, *Necator* and *Strongyloides*).

## Discussion

The present study investigated the association between dietary intake from complementary feeding, intestinal barrier function and EE in Brazilian children. Our results showed that, besides enteropathogen burden and SES, the lower intake of energy and dietary fibre were associated with worsened gut barrier function and higher intestinal inflammation, respectively. To our knowledge, this is the first study to assess the association between these variables in a Brazilian cohort of children.

Globally, inadequate nutrient intakes have been declining over the past 50 years, but it is still a reality in some low- and middle-income countries^(56–58)^. Cohort studies conducted in Bangladesh and Nepal found that the children had poor nutrient adequacies^(57,58)^. In these cohorts, the dietary intake of most of the vitamins, such as vitamins A, E and the B complex, and minerals, such as Ca, Fe and Zn was insufficient compared with international recommended values^(57,58)^. On the other hand, our children had sufficient intake for most of the nutrients analysed. Fibre intake was the only one to show poor adequacy. SES is a determinant variable for food intake. As shown in a previous report from the MAL-ED study^(37)^, the WAMI index from Brazilian children (0·8) was higher than those from Bangladesh (0·55) and Nepal (0·69). Therefore, the differences in the nutritional adequacies between sites may be explained by the socio-economic context.

Few studies in the last decade have assessed the dietary intake in children from Brazil, using the adjustment of habitual nutrient intake by the intra-individual variability,^(59–64)^ and all of the studies were conducted using a cross-sectional design. These studies have shown that the energy, macro-, or micronutrients habitual intakes of Brazilian children were adequate and higher than the recommended by age^(63,64)^. From these studies, only two assessed children on a comparable age to our study population. Both found an adequate energy intake and higher intake than the requirement for most of the micronutrients evaluated,^(63,64)^ as shown in the present study. However, one study found inadequate intake of Fe and Zn^(63)^, and the other study found inadequacies for Fe, vitamin E and folate^(64)^. These differences in intakes may be attributed to the characteristics of the population evaluated and its food access and consumption, which vary significantly according to regions in the Brazilian country.

This scenario of the habitual nutrient intake from complementary feeding with adequate or high-energy and nutrient intake values but low in fibre intake may be due to increased consumption of industrialised infant foods in Brazil. Many of these foods, although fortified with vitamins and minerals, are also rich sources of food additives and considered ultra-processed foods^(62,65)^. Recent data from studies conducted in Brazilian children have shown that the introduction of ultra-processed foods occurs before the 6th month of life, and about 6 months, the prevalence of ultra-processed foods intake is already 43 %^(65–67)^. In our study population, most of the mothers offering milk other than breast milk from the first month of life did it as a combined preparation with wheat, oat and rice-based industrialised powder product as previously described^(68)^. These products are examples of ultra-processed fortified foods which present low fibre content, that were regularly found in the 24-h records from 9 to 15 months. This high consumption of ultra-processed foods in children under 2 years might be a matter of concern, given the negative outcomes associated with the consumption of these foods. Studies in adults have shown an association between the consumption of ultra-processed foods with increased all-cause mortality^(69)^, and increased risk of obesity^(70)^, CVD^(71)^ and cancer^(72)^.

A national cross-sectional study with US children showed similar results to those found in the present study, with adequate micronutrient intake for most of the nutrients and low fibre intake, for young children^(73)^. The US population also has a high intake of ultra-processed foods^(74)^, and despite the socio-economic differences with Brazil, this intake may have contributed to the low nutrient inadequacies observed in both countries.

The median adequacy of protein intake was adequate. Although the lack of consensus, it has been proposed that excessive protein intake in infancy would increase plasma concentrations of insulin-releasing amino acids, stimulating insulin and insulin-like growth factor 1 secretion and increasing body weight gain and fat deposition, as well as the subsequent increased risk of obesity, adiposity and chronic non-communicable diseases^(75,76)^. Nevertheless, protein requirements are based on the minimal amount of protein needed to deliver the minimal amount of essential amino acids to prevent deficiency^(44)^. Therefore, the high intakes found in our population might not imply risks, and further studies should address that.

We found negative correlations between energy adequacy and L:M ratio and between folate NAR and NEO concentrations, whereas there was a positive correlation between thiamine NAR and MPO, and between Ca NAR and NEO concentration. Although current pathways appear unclear, thiamine, folate and Ca have been associated with intestinal barrier function and protection against intestinal inflammation^(76–79)^. The depletion of thiamine impairs the initiation of IgA antibody responses against oral antigens^(77)^. Folate deficiency decreases resistance to infections and increases susceptibility to intestinal inflammation (impairing the function of dendritic cells)^(78)^. Ionic calcium extra and intracellular concentrations influence intestinal barrier function and immunity^(79,80)^. Despite these relationships observed in the bivariate analysis, the results of the multiple linear regression final models showed that the nutritional components that were associated with the biomarkers were energy and fibre.

Recent data from the MAL-ED cohort study show that the levels of intestinal inflammation represented by intestinal neutrophil activity (MPO) and Th1 activity (NEO), and protein waste (AAT) are increased and associated with the presence of EE in infants and young children^(27)^. In the present study, all three biomarkers of EE had high median concentrations relative to published references for healthy individuals in high-income country settings,^(81–83)^ thus suggesting the presence of EE among our study population. Additionally, Lima *et al.*^(84)^ found that children from the MAL-ED case–control study the values of the L–M test, and MPO and NEO were elevated in both malnourished and nourished Brazilian children. Parasites and viruses were the most present pathogens in our population. Bacteria significantly contributed to intestinal permeability. A recent murine model for *Shigella* infection has shown that Zn deficiency enabled persistence of infection, enteropathy and stunting^(85)^. These data suggest that pathogens may exhibit specific interactions with diet deficient in particular nutrients, and further work exploring these interactions will be conducted.

Our findings illustrate that in a context of macro- and micronutrient sufficiency, there are associations between energy adequacy and intestinal barrier function, and between fibre adequacy and EE. Most of the accumulated knowledge about the role of energy intake on the intestinal barrier comes from studies with animal models using energy–protein-restricted diets. These diets are associated with changes in the structure and function of the intestinal epithelium, increased intestinal permeability, and induction of intestinal and systemic immune responses^(86–89)^. Human studies focus on the impact of nutritional status (which reflects protein-calorie restriction) on the function of the intestinal barrier^(88)^. Growth deficit, a characteristic of protein–energy restriction in children, has been associated with increased intestinal permeability assessed by the L:M ratio in children with EE in Bangladesh^(90)^, Malawi^(91)^, India^(92)^ and in Brazil^(84,93)^.

Interestingly, a cross-sectional study among children with 18 months in Bangladesh found that energy intake from complementary foods was inversely associated with L:M ratio^(94)^. Children in Bangladesh have suboptimal dietary energy intake as well as intakes of carbohydrate, fat and protein^(95)^. Thus, these results and our data corroborate with that the energy intake seems to have a protective effect on the gut barrier function.

In the present study, the adequacy of fibre intake was inversely associated with the EE score, indicating a beneficial effect of the adequate intake of fibres from the complementary diet on faecal biomarkers of intestinal inflammation. Dietary fibres function may explain this association between the intake of dietary fibres and improvement of the intestinal barrier and inflammation through the production of SCFA by the intestinal microbiota^(15)^ as well as acting directly on mucosal components^(7)^. SCFA have beneficial effects on the intestinal barrier by acting as an energy source for enterocytes. These nutrients also have anti-inflammatory properties via inhibition of the activation of NF-κB and by binding to G-protein coupled receptors, GPR41, GPR43 and GPR109A^(96,97)^. On the other hand, some fibres can interact directly with immune cells, attenuating the inflammatory cytokines in dendritic cells by modulating intestinal epithelial cells^(7)^. This pathway could be related to the improvement of the EE score, a result found in the present study, since the dendritic cells release NEO, consequently to the activation of Th1 lymphocytes.

Nutrient variations above recommendations did not appear to be related to L–M or EE in our study. Variations in nutrient intake below the recommendations might be associated with EE, such as the case of fibre, and studies in children with nutrient-deficient diets could observe these associations.

Some potential limitations of our study should be taken into consideration. Both random and systematic errors can affect the estimate of dietary intake. To minimise this bias, trained interviewers conducted the 24-h recalls using standardised methods^(34)^, and they were also trained to establish good communication with the participants to minimise the influence of psychological determinants of misreporting^(98)^. In addition, the residual method of energy adjustment was used to decrease the influence of misreporting energy intake, once this method allows the amounts of nutrients to be independent of total energy intake^(42,98)^.

Nevertheless, our results showed that, for most of the evaluated nutrients, the intakes were adequate, even higher than the recommended. Our primary interest was to assess the habitual intake of energy and nutrients from the food of the complementary feeding. Analysing only the nutrient from complementary feeding could have underestimated the results considering that 61·5 % of the children received breast milk at 15 months of age. However, we have shown that MAR (an indicator of the overall nutritional quality of the diet) was high, the NAR was also high for all micronutrients and the median energy daily intake achieved the recommended values. Interestingly, in the present study, we did not find any associations of breast-feeding type or duration and the dependent variables analysed (not shown). This result could be due to the low intake of breast milk by the age of 15 months when the gut biomarkers were assessed in the present study.

The key strength of the study is the longitudinal design with monthly measurements of nutrient intake, enabling to remove intra-individual variation, which allowed a more reliable assessment of habitual nutrient intake than in a cross-sectional design. Although the data found are not nationally representative, and rigorous sampling was not performed, the findings may reflect those of most urban *favelas*, since our community study represents a typical urban *favela* from the northeast, as previously described by Lima *et al.*^(33)^. Besides, the present study is the first in Brazil that collected longitudinal information on infant feeding practices and analysed data collected from home visits performed twice a week and monthly. Longitudinal data collection allowed a rigorous classification of breast-feeding practices and habitual nutrient intake, and prospective national studies using a similar methodology for analysis and data collection are needed.

In conclusion, the data add to the knowledge that beyond enteropathogens, other modifiable factors, such as diet may be associated with the intestinal barrier function and EE. We found inverse associations between energy intake from complementary food and L:M ratio and between fibre intake and EE score. These results suggest a protective effect between adequate energy intake on the intestinal barrier function and a beneficial effect of the intake of fibres from the complementary diet to prevent EE in children.
